# Characterization of Monoclonal and Polyclonal Antibodies Recognizing Prostate Specific Antigen: Implication for Design of a Sandwich ELISA

**Published:** 2019

**Authors:** Sahar Raoofi Mohseni, Forough Golsaz-Shirazi, Mostafa Hosseini, Jalal Khoshnoodi, Tannaz Bahadori, Mohammad Ali Judaki, Mahmood Jeddi-Tehrani, Fazel Shokri

**Affiliations:** 1. Department of Immunology, Faculty of Public Health, Tehran University of Medical Sciences, Tehran, Iran; 2. Department of Epidemiology and Biostatistics, Faculty of Public Health, Tehran University of Medical Sciences, Tehran, Iran; 3. Monoclonal Antibody Research Center, Avicenna Research Institute, ACECR, Tehran, Iran

**Keywords:** ELISA, Monoclonal antibodies, Prostate cancer, Prostate specific antigen

## Abstract

**Background:**

Prostate cancer is the second most common cancer in men. Prostate-Specific Antigen (PSA) is a tumor-associated glycoprotein with enzymatic activity which is secreted by the prostate gland. Following entry to the blood, 70–90% of PSA forms complexes with protease inhibitors and its enzymatic activity is inhibited. The serum level of PSA is increased and the rate of free PSA (fPSA) to total PSA is decreased in prostate cancer patients. Therefore, measurement of PSA and fPSA in serum is very valuable for diagnosis and prognosis of prostate cancer.

**Methods:**

In the present study, five anti PSA monoclonal Antibodies (mAb) were characterized by Enzyme-Linked Immunosorbent Assay (ELISA) and immunoblotting. For designing a sandwich ELISA, epitope specificity of these antibodies was studied by a competition ELISA. Free PSA was purified by electroelution technique from seminal plasma and used to produce polyclonal anti-fPSA antibody in rabbit. Purified polyclonal antibody (pAb) and mAbs were conjugated with HRP enzyme and Biotin (Bio) to set up the sandwich ELISA.

**Results:**

Three of the mAbs were found to recognize PSA similarly. One of these mAbs (2G3) was paired with anti-fPSA pAb to detect fPSA in serum. Eventually, serum fPSA concentration of 356 subjects was measured and compared by our designed ELISA and a commercial ELISA kit. Our results demonstrated a significant correlation (r=0.68; p<0.001) between the two assays. Sensitivity and specificity of our designed ELISA was 72.4 and 82.8%, respectively.

**Conclusion:**

These results imply suitability of our designed ELISA for detection of fPSA in patients with prostate cancer.

## Introduction

Prostate Cancer (PCa) is one of the most common cancers and also is the sixth leading cause of cancer death in men globally 1. Prostate-Specific Antigen (PSA), a 30–33 *kDa* androgen-dependent serine protease and a tissue Kallikrein family member, is the most valuable tumor marker for diagnosis, monitoring, and screening of PCa 2–4. Initially, this antigen is produced from prostatic ducts and acinar epithelium then secreted into the lumen 5, where it cleaves semenogelin in the seminal coagulum 6. As an important protein of seminal plasma (SP), the function of PSA in prostate is to liquefy the sperm-entrapping seminal coagulum after ejaculation 7. 70–90% of PSA that enters to the bloodstream makes a complex with a protease inhibitor called alpha-1-anti-chymotrypsin (PSA-ACT). A small amount of PSA forms complexes with other protease inhibitors, including alpha-2-macroglobulin (PSA-AMG) and alpha-1-anti-trypsin and protein C. 10–30% of the total PSA in serum is not bound to prostate inhibitors or proteins and is called free PSA (fPSA) 8.

Different studies in the early 1990s showed that total PSA in the serum could be used for identification of patients with prostate cancer 9. In healthy males, PSA is normally released into the blood at low concentrations (<4 *ng/ml*) 10. During the early stage of prostate cancer development, disruption of the cell membrane and basal membrane allows PSA to leak in to the bloodstream, resulting in elevated serum levels of PSA 11. Presence of higher levels of PSA (>4 *ng/ml*) is one of the important indicators of PCa 12. However, increased level of serum PSA could also be attributed to Benign Prostatic Hyperplasia (BPH) and prostatic disease, causing false positive screening results 13. In order to improve specificity and sensitivity of PSA test for early detection of PCa, some strategies, such as measuring PSA velocity (rate of change in serum PSA level over time), PSA doubling time (PSA dynamics), and PSA density (adjusting the level of PSA to the size of the prostate gland) have been proposed 14. The ratio of free (unbound) PSA to total PSA in men with prostate cancer is lower than that in men with BPH. The lower the ratio is, the greater the probability of prostate cancer. Therefore, instead of only reporting PSA, calculating the ratio of fPSA to total PSA and determining the level of fPSA can improve the sensitivity and specificity of PSA test 15.

Given the importance of high affinity antibodies against PSA with the ability to detect low levels of fPSA in serum, the purpose of this study was to characterize new anti-PSA antibodies and designing a new sensitive sandwich ELISA to measure fPSA in human serum and compare its sensitivity with that of a commercial kit.

## Materials and Methods

### Use of animals

Animal experimentation was complied with local and national regulations, including the Iranian Ministry of Health guidelines for the welfare of animal research.

### Handling human tissue

Collection and handling human serum was carried out according to regulations of Tehran University of Medical Sciences. This study was approved by the ethical committee of Tehran University of Medical Sciences.

### Free PSA purification and characterization by SDS-PAGE and Western blotting

The seminal fluid samples were initially centrifuged at 350 *g* for 10 *min* to remove cells and clarify the plasma. The collected plasma was then ultracentrifuged at 36000 *g* for 20 *min* at 4°*C* and the supernatant was stored at −20°*C*.

PSA was purified from seminal plasma by electroelution technique. The clarified plasma was dialyzed against Phosphate-Buffered Saline (PBS) for 2 *hr* at RT. Seminal plasma obtained from this stage was boiled for 5–10 *min* at 100°*C* and separated on 12% polyacrylamide gel. After electrophoresis, the polyacrylamide gel was reverse stained by imidazole 0.2 *M* and ZnCl2 0.2 *M*. The fPSA bands (30–33 *kDa*) were cut, fragmented to small slices and extracted from the gel by electroeluter (model 422 Electro-Eluter, Life science research, Bio-Rad) in two stages. The sample was transferred to the sample chamber with running buffer for 4 *hr* at 10 *mA* current. Samples were collected from the chamber, and dialyzed against PBS overnight. The purity of purified fPSA samples was confirmed by sodium dodecyl sulfate-polyacrylamide gel (SDS-PAGE) and Western blotting. The total protein concentration was measured using BCA protein assay kit (pierce BCA assay kit, Thermo Fisher Scientific, USA).

After electroelution, two micrograms of purified fPSA was analyzed on 12% SDS-PAGE under non-reducing condition. After electrophoresis, antigens were transferred to the nitrocellulose membranes. After blocking with 5% skim milk in PBS containing 0.05% Tween 20 (PBST) at 4°*C* overnight, the membrane was incubated with 2G3-HRP for 1.5 *hr* at RT. After washing, blotted membrane was developed with enhanced chemiluminescence (ECL) detection system (Amersham, GE Healthcare, USA).

### Production, purification and characterization of polyclonal anti fPSA antibody

One New Zealand white rabbit was intramuscularly immunized with 40 *μg* of purified fPSA emulsified in Freund’s complete adjuvant. Additional injections of 20 *μg* fPSA in incomplete Freund’s adjuvant (IFA) were followed biweekly. During immunization intervals, the serum specific antibody titer was tested by ELISA.

In order to purify specific anti-fPSA antibodies from serum of the immunized rabbit, an affinity purification column was prepared. Purified fPSA was coupled to CNBr-activated Sepharose 4B (GE Healthcare, Stockholm, Sweden) according to the manufacturer's protocol. Serum of the immunized rabbit was clarified by centrifugation and diluted 1:2 with PBS and passed through the column. After washing the column with PBS, antibodies were eluted with Gly-HCL 0.2 *M*, pH=2.5. Eluted anti-fPSA antibodies were immediately dialyzed against PBS. The specificity of purified rabbit polyclonal anti-fPSA was confirmed by ELISA and Western blotting.

10 *μg* PSA (90% PSA-ACT, 10%fPSA), 2 *μg* fPSA (purified fPSA) and 2 *μg* ACT (Sigma Aldrich) were electrophoresed on 12% SDS-PAGE under reducing and non-reducing conditions. The antigens were transferred to nitrocellulose membrane and blotted membrane was blocked with 5% skim milk-PBST at 4°*C* overnight. The membrane was incubated with appropriate dilution of rabbit anti-PSA-HRP for 1.5 *hr* at RT. After washing, the blots were developed with ECL detection system.

### Assessment of cross-reactivity of mouse monoclonal and rabbit polyclonal antibodies with recombinant human kallikrein-2 (rhk2)

Important concern with antibodies against fPSA is cross-reactivity with other members of the kallikrein family, especially human glandular kallikrein (hK2). Therefore, the cross-reactivity of anti-PSA monoclonal and polyclonal antibodies with rhk2 was evaluated by indirect and sandwich ELISA.

For indirect ELISA, 2.5 *μg/ml* rhk2 (R&D, USA) and 5 *μg/ml* fPSA (NIBSC, UK) were coated on 96-well plate (Maxisorp, Nunc, Denmark) and incubated at 37°*C* for 1.5 *hr*. The plate was washed 3 times with PBST and blocked with 3% skim milk-PBST for 1 *hr* at 37°*C*. After washing, 5 *μg/ml* of each anti-PSA mAbs 2G2, 2G3, 2D6, 2C8 16 and 1E11 (A gift from Professor Abbas Ghaderi, Cancer Research Institute, Shiraz, Iran) and also purified rabbit anti-fPSA polyclonal antibody (Rα-fPSA) were added to the plate for 1 *hr* at 37°*C*. HRP-conjugated rabbit anti-mouse immunoglobulin and HRP-conjugated sheep anti-rabbit antibody were added and incubated for 1 *hr* at 37°*C*. After washing, 3,3’,5,5’Tetramethylbenzidine (TMB) substrate solution (Pishtazteb, Tehran, Iran) was added and the reaction was stopped by addition of HCL 1N and the OD was measured at 450 *nm* by ELISA reader (BioTeK Power Wave XS, USA).

The reactivity of these antibodies with rhk2 and fPAS was also checked by sandwich ELISA. 5 *μg/ml* of each anti-PSA mAb (2G2, 2G3, 2D6, 2C8, and 1E11) was coated on a 96-well plate (Maxisorp, Nunc, Denmark) and incubated at 37°*C* for 1.5 *hr*. The plate was washed with PBST and wells were blocked with 3% skim milk-PBST for 1 *hr* at 37°*C*. fPSA (NIBSC) and recombinant hk2 were serially diluted from 2 *μg/ml* and added to the respective wells and then the plate was incubated at 37°*C* for 1 *hr*. HRP-conjugated polyclonal rabbit anti-fPSA diluted to the 1/500 was added and incubated for 1 *hr* at 37°*C*. After washing, TMB substrate was added and the reactivity was stopped and the OD was measured as explained above.

### Assessment of overlapping epitopes recognized by anti-PSA mAbs

Competition ELISA was performed to evaluate the overlapping epitopes recognized by different anti-PSA mAbs. Three monoclonal antibodies (2G2, 2G3, 2D6) were conjugated with biotin. 96-well plates (Maxisorp, Nunc, Denmark) were coated with seminal plasma diluted 1/1000 in PBS for 1.5 *hr* at 37°*C*. After washing with PBS-T, plate was blocked with 3% BSA-PBST for 1 *hr* at 37°*C*. Varying concentrations (0.1–40 *μg/ml*) of competing mAbs (2G2, 2G3, 2D6) and 2.5 *μg/ml* of biotinylated mAbs were added to each well and allowed to compete with each other for binding to fPSA for 1 *hr* at 37°*C*. After washing, 1/5000 dilution of streptavidin-HRP prepared in blocking buffer was added and incubated at 37°*C* for 1 *hr*. Following washing, TMB substrate solution was added and the reaction was stopped with 1N HCL. Finally, the OD was measured at 450 *nm* by ELISA reader. Inhibition percentage was calculated as follows:

$$\%\text{Inhibition}\;=\left[\left({\text{OD}}_{\text{Without}\;\text{competitor}}-{\text{OD}}_{\text{with}\;\text{competitor}}\right)/{\text{OD}}_{\text{Without}\;\text{competitor}}\right]\times 100$$

### Identification of appropriate capture and detector antibodies

In order to design a sensitive ELISA to measure serum PSA, the reactivity of 2G3 anti-PSA mAb and polyclonal rabbit anti-fPSA in coating and detection phase was evaluated. Therefore, 10 *μg/ml* of each antibody was separately coated in ELISA plate and incubated at 37°*C* for 1.5 *hr*. The plate was washed with PBST and blocked with 3% BSA-PBST at 37°*C* for 1 *hr*. Serially diluted fPSA (NIBSC) ranging from 125 *ng/ml* to 1.9 *ng/ml* was added to the plate and incubated at 37°*C* for 1 *hr*. After washing, 2 *μg/ml* biotin-conjugated antibodies (2G3-biotin and rabbit anti-fPSA-biotin) were added to the relevant wells. Streptavidin-HRP conjugate at a dilution of 1/1200 in the blocking buffer was added to the wells after washing and incubated at 37°*C* for 1 *hr*. After washing and adding TMB substrate solution, reactivity was stopped as explained previously and OD was measured at 450 *nm*.

### Evaluating the specific reactivity of 2G3-HRP and rabbit anti-fPSA-HRP by Western blotting

Specific activity of HRP-conjugated 2G3 and rabbit anti-fPSA antibodies were confirmed with Western blotting. 100 *ng* PSA (90% PSA-ACT, 10% fPSA-NIBSC) and 500 *ng* fPSA (NIBSC) were subjected to 12% SDS-PAGE under non-reducing condition and transferred to nitrocellulose membranes. After blocking with 5% skim milk-PBST at 4°*C* overnight, the membranes were incubated with appropriate dilution of 2G3-HRP or rabbit anti-fPSA-HRP for 1.5 *hr* at RT. After washing and adding substrate, the blots were developed with ECL detection system.

### Comparison of detection limit, specificity and sensitivity of designed ELISA with a commercial sandwich ELISA kit

ELISA plate wells were coated with 10 *μg/ml* polyclonal rabbit anti-fPSA at 4°*C* overnight. The plate was washed 3 times with PBS-T and blocked with BSA 1%. Different concentrations of fPSA and TPSA antigens (NIBSC) were prepared in BSA1% and added to the plate and incubated at 37°*C* for 1 *hr*. After washing, biotin-conjugated 2G3 mAb (5 *μg*) diluted in BSA1% was added and incubated for 15 *min* at 37°*C*. After adding appropriate dilution of streptavidin-HRP in BSA1% and incubating for further 20 *min* at 37°*C*, wells were washed and TMB substrate solution was added. The reaction was stopped with 1N HCL and the OD was measured at 450 *nm* by ELISA reader. This experiment was also performed in parallel using fPSA commercial kit (CanAg, Gothenburg, Sweden) according to the kit's protocol.

In order to determine specificity and sensitivity of our designed ELISA, serum samples of 356 subjects, collected from three medical diagnostic laboratories anonymously, were tested by our designed sandwich ELISA and the commercial kit and the results were compared with each other.

### Assay validation and statistical analysis

Data were analyzed using STATA statistical software (version 14.0). Pearson correlation test was used to evaluate the correlation between our designed EL-ISA and commercial kit. In order to examine diagnostic value of designed ELISA, ROC curve was drawn. The Area Under the Curve (AUC) with 95% Confidence Interval (CI 95%) was calculated in order to predict accuracy and determine the cut off value. p-values of <0.05 was considered statistically significant.

## Results

### Purification of free PSA from seminal plasma

**Figure 1.**
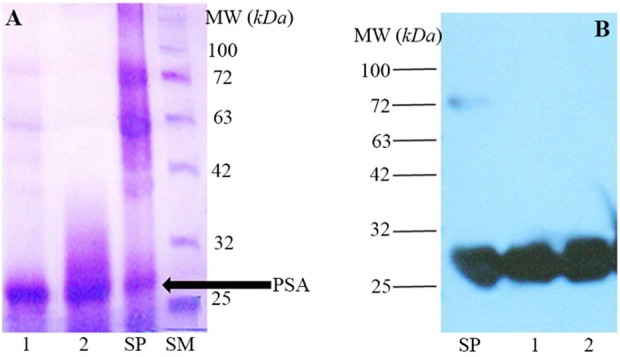
A) SDS-PAGE analysis of Seminal Plasma (SP) and purified fPSA (lanes 1 and 2), SM: Size marker. B) Western blot analysis of SP and purified fPSA (lanes 1 and 2). 2G3 mAb conjugated with HRP were added to blotted membrane.

4.4 *mg* of fPSA was purified from seminal plasma by electroelution. Purity of the purified fPSA was analyzed by SDS-PAGE (Figure 1A) and Western blotting (Figure 1B).

### Production, purification, and characterization of polyclonal anti fPSA antibody

**Figure 2.**
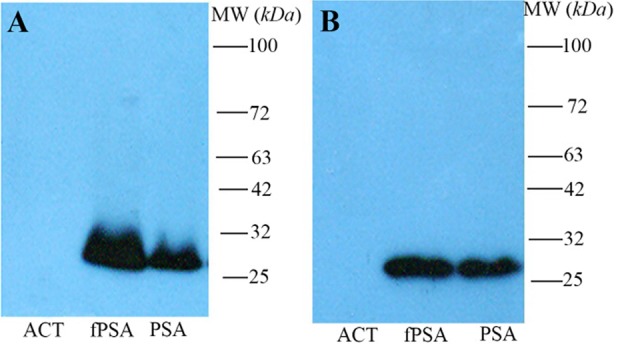
Western blot analysis of polyclonal rabbit anti-fPSA. Non-reduced (A) and reduced (B) antichymotrypsin (ACT), fPSA and total PSA (90% PSA-ACT, 10% fPSA) proteins were subjected to SDS-PAGE, transferred to a nitrocellulose membrane and reveled with HRR conjugated rabbit anti-fPSA.

Sera from immunized rabbit were titrated on fPSA pre-coated ELISA plates. The results indicate that after five immunizations, the specific anti-fPSA titer reached plateau. Anti-fPSA specific antibody was purified from hyperimmunized rabbit serum by affinity chromatography. Results of Western blotting showed that the purified antibody specifically recognized fPSA at reducing and non-reducing conditions (Figure 2).

### Assessment of reactivity of mouse monoclonal and rabbit polyclonal antibodies by indirect and sandwich ELISA

Reactivity of the anti-PSA antibodies was evaluated by indirect and sandwich ELISA. While all the mAbs (2G2, 2G3, 2D6, 1E11, and 2C8) as well as the pAb were able to specifically recognize fPSA by indirect ELISA, only 3 of the mAbs (2G2, 2G3 and 2D6) were able to detect fPSA by sandwich ELISA. None of the mAbs reacted with rhk2, whereas the pAb reacted with rhk2 (Table 1).

**Table 1.** Reactivity of monoclonal and polyclonal anti-PSA antibodies with fPSA and human kallikrein 2

	**Indirect ELISA**		**Sandwich ELISA**	

**mAb/pAb**	**Detection of rhk2**	**Detection of fPSA**	**Detection of rhk2**	**Detection of fPSA**
**2C8**	−	+	−	−
**1E11**	−	+	−	−
**2G2**	−	+	−	+
**2G3**	−	+	−	+
**2D6**	−	+	−	+
**RbαPSA**	+	+	+	+

### Assessment of overlapping epitopes recognized by anti-PSA monoclonal antibodies

**Figure 3.**
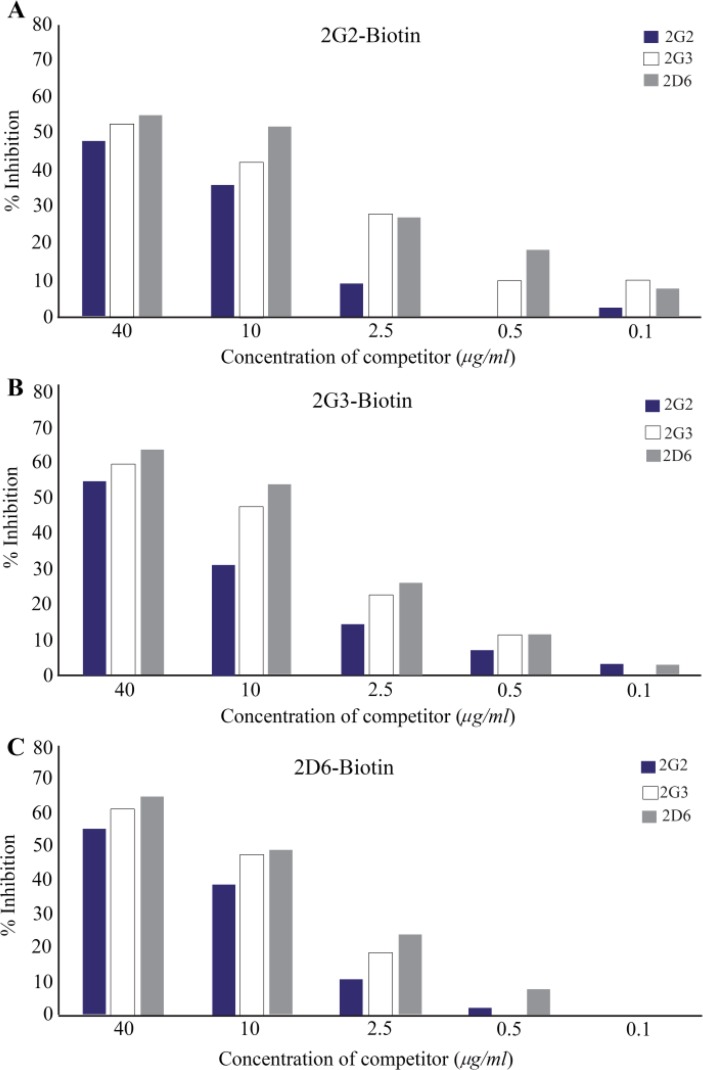
Competitive binding of monoclonal antibodies to free PSA. Competition ELISA was performed to evaluate the overlapping epitopes recognized by different anti-fPSA mAbs. Varying concentrations (0.1–40 *μg/ml*) of unconjugated mAbs (2G2, 2G3, 2D6) (competitor) and 2.5 *μg/ml* of biotinylated mAbs were added separately to fPSA precoated wells and allowed to compete with each other for binding to fPSA. As the concentration of competitor antibody increases, the percentage of inhibition increases, which indicates that the competing antibodies and biotinylated antibodies recognize similar epitopes or epitopes in close proximity. Figures A, B and C represent binding inhibition of the three mAbs.

Varying concentrations (0.1–40 *μg/ml*) of competing mAbs (2G2, 2G3, 2D6) together with 2.5 *μg/ml* of biotinylated mAbs were added to fPSA-precoated plate and allowed to compete with each other for binding to fPSA. The results of competition ELISA showed that 2G3, 2G2, and 2D6 mAbs recognize either the same epitope or epitopes located very close to each other (Figure 3). Since all mAbs displayed the same pattern of reactivity, one of the mAbs (2G3) was selected for the design of sandwich ELISA.

### Assessment of binding of 2G3 mAb and anti-PSA pAb to free and complexed PSA

**Figure 4.**
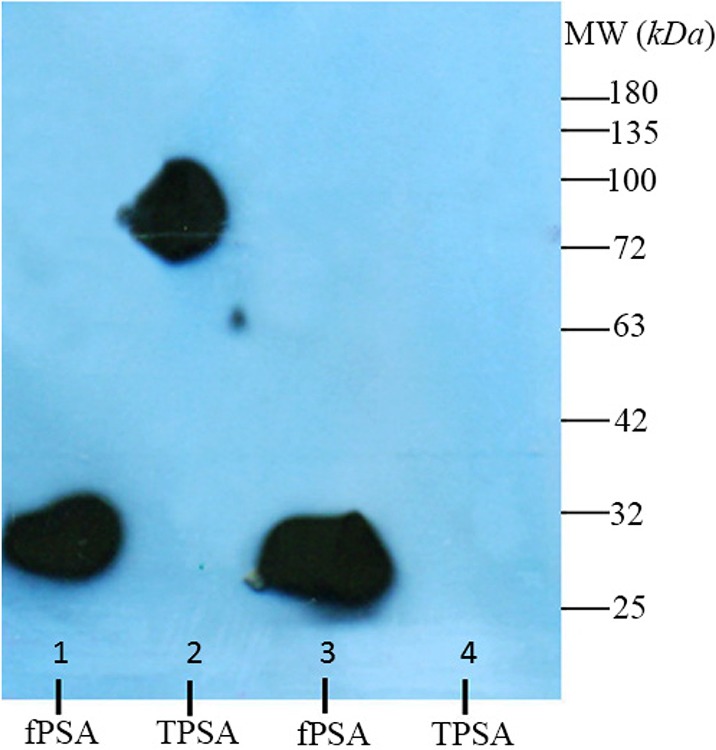
Western blotting analysis of reactivity of monoclonal (2G3-HRP) and polyclonal rabbit anti-fPSA-HRP antibodies with fPSA and TPSA (90% PSA-ACT, 10% fPSA). The results showed that 2G3 is able to recognize fPSA and PSA-ACT complex in TPSA. fPSA: free PSA, TPSA: 90% PSA-ACT, 10% fPSA. lanes 1 and 2: 2G3-HRP; lanes 3 and 4: polyclonal rabbit anti-fPSA–HRP.

Western blot was performed using fPSA and total PSA (TPSA) which consists of 90% PSA-ACT and 10% fPSA. The results indicate that HRP-conjugated rabbit anti-fPSA could only detect fPSA (∼30 *kDa*), but not PSA-ACT (∼90 *kDa*). However, HRP-conjugated 2G3 specifically recognizes both fPSA and PSA-ACT (Figure 4). The level of TPSA which was run in this experiment was apparently not high enough to allow detection of the residual fPSA in this preparation, taking in to consideration the detection limit of our ECL system.

### Identification of appropriate capture and detector antibodies for sandwich ELISA

**Figure 5.**
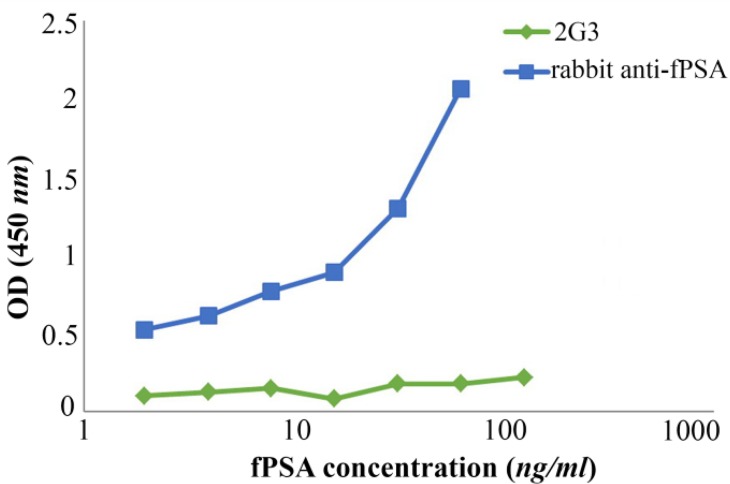
Assignment of the capture and detector antibodies in the designed sandwich ELISA. Wells of ELISA plate were coated separately with rabbit anti-fPSA pAb and 2G3 mAb. After adding different concentrations of fPSA, biotinylated-2G3 and rabbit anti-fPSA, respectively, were added as detector. Green line: 2G3 mAb used as coating antibody and biotinylated-rabbit anti-fPSA used as detector antibody; Blue line: rabbit anti-fPSA pAb used as coating antibody and biotinylated-2G3 used as detector antibody.

The results show that using rabbit anti-fPSA pAb as capture antibody and 2G3 mAb as detector antibody makes the assay more sensitive (Figure 5).

### Assay validation and statistical analysis

**Figure 6.**
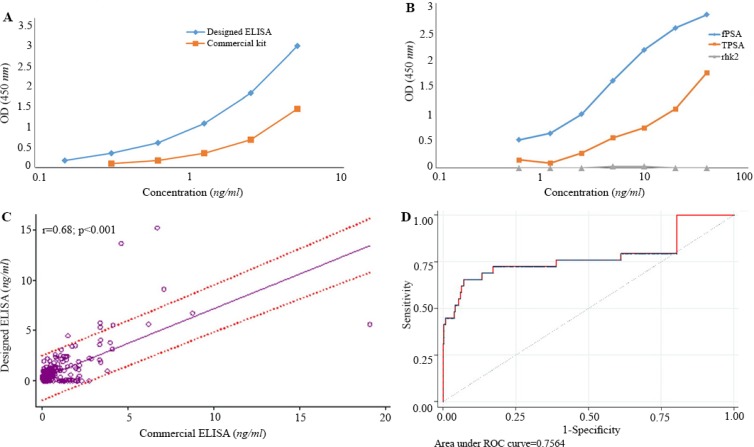
A) Standard curves obtained for the designed ELISA and commercial kit. Red and blue lines represent standard curves for commercial kit and designed ELISA, respectively. B) Assessment of cross-reactivity of designed ELISA with rhk2. TPSA contains 10% fPSA, as the diagram shows OD of 40 *μg/ml* of TPSA that contains 4 *μg/ml* fPSA is similar to that of 5 *μg/ml* pure fPSA. Our results showed that designed ELISA specifically recognizes fPSA. C) Pearson correlation analysis of the results obtained by the designed ELISA and commercial kit. As the result show there is a significant correlation between the two assays. D) ROC Curve analysis of the results obtained by the designed ELISA and commercial kit. The best Cut off point for designed ELISA is 0.98 *ng/ml*.

Standard curves were constructed for our designed ELISA and the commercial sandwich ELISA kit. Using a standard fPSA preparation obtained from the NIBSC (Figure 6A), down to 0.156 *ng/ml* of fPSA was detected by both assays.

Reactivity of our designed ELISA and also the commercial CanAg kit with fPSA, TPSA, and rhk2 was checked. The results showed that the commercial CanAg kit does not show cross-reactivity to even high concentrations (40 *ng/ml*) of rhk2; likewise, the designed ELISA specifically recognizes fPSA with no cross-reactivity to rhk2 (Figure 6B).

Specificity and sensitivity of the designed ELISA were determined by measuring the concentration of fPSA in serum of 356 subjects and comparing the results with those of the commercial kit. A significant positive correlation was noted between the two assays (r=0.68, p<0.001) (Figure 6C).

Based on these results and the ROC analysis (Figure 6D) and the best cutoff point, sensitivity and specificity of our designed assay were found to be 72.4 and 82.8%, respectively.

Assay variation was checked using 3 different serum samples with different concentrations. Each sample was tested 5 times to calculate the Coefficient of Variation (CV). Intra-assay and inter-assay CV were found to be 2.93–16.42% (mean 9.67%) and 16.43–21.48% (mean 18.95%) for the designed ELISA.

## Discussion

In this study, a panel of 5 mAbs and one pAb which were raised against fPSA purified from normal seminal plasma were characterized. Some of these monoclonal antibodies have been previously characterized 16; however, this is the first time that these antibodies were used to design a sandwich ELISA. All these antibodies bind to fPSA in an antigen based indirect ELISA; however, two of the mAbs (2C8 and 1E11) failed to recognize the antigen by sandwich ELISA (Table 1). Epitope specificity of the three positive mAbs was investigated by a competition ELISA. The results indicate recognition of the same epitope or closely related epitopes by these mAbs. Thus, one of these mAbs (2G3) was selected for further characterization and design of a diagnostic sandwich ELISA. This mAb and anti-PSA pAb were analyzed by immunoblotting for binding to fPSA and complexed PSA. The results demonstrated that while 2G3 mAb recognizes an epitope shared by both free and complexed PSA, the pAb binds to an epitope unique to fPSA (Figure 4).

Taking these data in to account, an attempt was made to design and set up a sandwich ELISA for detection and measurement of fPSA. In contrast to indirect ELISA, which usually shows low level of cross-reaction, in sandwich ELISA some monoclonal antibodies might show cross-reaction with polyclonal antibodies that have been raised in other species. Therefore, paired antibodies should be assessed to identify the appropriate capture and detector antibodies. Surprisingly, contrary to our expectation, 2G3 mAb did not perform well in the solid phase (Figure 5). Therefore, the pAb was employed in the solid phase and biotinylated 2G3 mAb was used in the liquid phase as the detector antibody.

The designed ELISA was found to be sensitive detecting down to 0.2 *ng/ml* fPSA (Figure 6A). Our designed ELISA (Figure 6B) and also commercial CanAg kit did not show cross-reaction to rhk2. In the next step, 356 serum samples were tested by our designed assay as well as the commercial sandwich ELISA kit in parallel. The intra- and inter-assay variations were 9.7 and 18.9%, respectively, indicating reasonable reproducibility of this assay. The sensitivity and specificity of the designed assay were 72.4 and 82.8%, respectively. These results imply development of a reasonably efficient assay system for measurement of PSA. Most of the samples that were analyzed in this study contained low levels of fPSA. Due to this issue, the specific signals in our assay were low which could explain the moderate correlation with the commercial kit (r=0.68, p<0.001) (Figure 6C).

In 1999, Black *et al* developed and characterized 11 anti-PSA mAbs to design an ELISA test to measure fPSA level in the serum, and the designed ELISA was considered as the most sensitive fPSA assay reported in that date 17. Thereafter, other researchers made great efforts to design more sensitive ELISA 18,19.

Besides PSA, detecting a panel of other biomarkers, such as hk2, urokinase Plasminogen Activator (uPA) and its receptor (uPAR), TGFβ, IL6, IL6R, Ki-67, ERK5, and DRE may improve PCa diagnosis, staging, prognosis and monitoring 4.

Measurement of different molecular forms of PSA has increased the diagnostic accuracy of this test. TPSA refers to both fPSA (unbound form) and bound PSA (complexes with other proteins, mainly α-1-antichymotrypsin) 20. Measuring the ratio of fPSA to TPSA can improve the discrimination of prostate cancer from benign prostatic disease, particularly in patients with TPSA level ranging from 4 to 10 *ng/ml* 21. Therefore, a precise and accurate method for measuring TPSA and FPSA has an important role in diagnosis and treatment of PCa 22–24.

In this study, cross-reactivity of anti-PSA monoclonal and polyclonal antibodies with recombinant hk2 antigen was evaluated by indirect and sandwich ELISA. None of the five anti-PSA mAbs showed cross-reaction with hk2. However, as expected, the anti-fPSA polyclonal antibody cross-reacted with hk2, which is more likely due to the high homology between hk2 and PSA. Despite the high homology between hk2 and PSA, they show distinct activity. Both belong to the kallikrein protein family, show 78% amino acid homology, and are produced by the prostatic epithelium 25.

## Conclusion

In conclusion, characterization of the monoclonal and polyclonal anti-PSA antibodies allowed the design of a sensitive sandwich ELISA assay for detection of fPSA, which may be useful for diagnosis, monitoring, and screening of patients with PCa. Establishment of high affinity monoclonal antibodies specific for fPSA will enable improvement of the detection limit, sensitivity and specificity of this assay.
